# Investigation of age and smoking in NSCLC patients with uncommon EGFR mutations

**DOI:** 10.5578/tt.202402913

**Published:** 2024-06-12

**Authors:** Yosuke MAEZAWA, Manato TAGUCHI, Takeshi KAWAKAMI, Toshihide INUI, Shinichiro OKAUCHI, Takeshi NUMATA, Toshihiro SHIOZAWA, Kunihiko MIYAZAKI, Ryota NAKAMURA, Kesato IGUCHI, Takeo ENDO, Tohru SAKAMOTO, Hiroaki SATOH, Nobuyuki HIZAWA

**Affiliations:** 1 Divisions of Respiratory Medicine and Thoracic Surgery, Mito Medical Center, University of Tsukuba-Mito Kyodo General Hospital, Mito, Japan; 2 Division of Respiratory Medicine, Kobari General Hospital, Noda, Japan; 3 Division of Respiratory Medicine, University of Tsukuba Faculty of Medicine, Tsukuba, Japan; 4 Division of Respiratory Medicine, Tsukuba Memorial Hospital, Tsukuba, Japan; 5 Departments of Respiratory Medicine and Surgery, National Hospital Organization Mito Medical Center, Ibarakimachi, Japan; 6 Division of Respiratory Medicine, Ryugasaki Saiseikai Hospital, Ryugasaki, Japan

## Abstract

**ABSTRACT**

**
Investigation of age and smoking in NSCLC patients with uncommon EGFR mutations
**

**Introduction:** *In addition to the two common epidermal growth factor recep- tor (EGFR) mutations, there are many uncommon mutations. Due to the high number of uncommon types, as well as the rarity of patients, there is lack of information regarding patient demographics, especially age distribution and smoking status. Against this background, we conducted an analysis to clarify the background of patients with uncommon EGFR mutations, especially con- sidering their age distribution and smoking status.
*

**Materials and Methods:** *We retrospectively reviewed the medical records of non-small cell lung cancer (NSCLC) patients diagnosed in a multicenter clini- cal practice from 2002 to 2023. Patients included all cases of non-advanced and advanced NSCLC with uncommon EGFR mutations.
*

**Results:** *Information on 158 patients with uncommon EGFR mutation was col- lected. Median age was 72 years, with the age distribution showing that most patients were in their 70s. There was a significant difference between the proportion of patients aged up to 59 years and the proportion aged 75 years or older. In 88 patients with a smoking habit history, a significant correlation was found between smoking index and age. Among non-smokers, there was a peak between ages 70 and 74, which was older than the peak among smokers.
*

**Conclusion:** *Even in elderly patients and NSCLC patients with a history of smoking, although it is unclear whether EGFR mutation is common or uncom- mon, EGFR gene testing should be performed considering the possibility of these patients being EGFR-positive.
*

**Key words:** *Epidermal growth factor receptor; non-small cell lung cancer; uncommon mutation; age
*

**ÖZ**

**
Nadir görülen EGFR mutasyonları olan KHDAK hastalarında yaş ve sigara kullanımının araştırılması
**

**Giriş:** *İki yaygın epidermal büyüme faktörü reseptörü (EGFR) mutasyonuna ek olarak, pek çok mutasyon da vardır. Nadir görülen tiplerin çokluğu ve hastaların nadirliği nedeniyle, hasta demografik özellikleri, özellikle yaş dağılımı ve sigara içme durumu hakkında bilgi eksikliği bulunmaktadır. Bu arka plana dayanarak, nadir görülen EGFR mutasyonlarına sahip hastaların geçmişini, özellikle yaş dağılımlarını ve sigara içme durumlarını göz önünde bulundurarak açıklığa kavuşturmak için bir analiz gerçekleştirilmiştir.
*

**Materyal ve Metod:** *2002'den 2023'e kadar çok merkezli bir klinik uygulamada teşhis edilen küçük hücreli dışı akciğer kanseri (KHDAK) hastalarının tıbbi kayıtlarını retrospektif olarak inceledik. Hastalar, yaygın olmayan EGFR mutasyonları olan tüm ileri evre olmayan ve ilerlemiş KHDAK vakalarını içeriyordu.
*

**Bulgular:** *Yaygın olmayan EGFR mutasyonuna sahip 158 hasta hakkında bilgi toplandı. Ortalama yaş 72 idi ve yaş dağılımı çoğu hastanın 70'li yaşlarda olduğunu gösteriyordu. Elli dokuz yaşına kadar olan hastaların oranı ile 75 yaş ve üzeri hastaların oranı arasın- da anlamlı bir fark vardı. Sigara içme öyküsü olan 88 hastada sigara içme indeksi ile yaş arasında anlamlı ilişki saptandı. Sigara içme- yenler arasında 70 ile 74 yaşları arasında bir zirve vardı ve bu, sigara içenler arasındaki zirveden daha düşüktü.
*

**Sonuç:** *Yaşlı hastalarda ve sigara içme öyküsü olan KHDAK hastalarında bile, EGFR mutasyonunun yaygın mı yoksa nadir mi olduğu belirsiz olsa da, bu hastaların EGFR pozitif olma olasılığı göz önünde bulundurularak EGFR gen testi yapılmalıdır.
*

**Anahtar kelimeler:** *Epidermal büyüme faktörü reseptörü; küçük hücreli dışı akciğer kanseri; yaygın olmayan mutasyon; yaş
*

## INTRODuCTION

The epidermal growth factor receptor (EGFR) mutation was the first driver gene discovered for non-small cell lung cancer (NSCLC) (Attili) (John). Among EGFR mutations, *Ex19* deletion and *Exon 21 L858R* are two of the most common mutations, and there are multiple uncommon mutations (1,2). Among the uncommon mutations, *G719X*, *L861Q*, and *S768I* mutations are relatively frequent (1,2). Therefore, there have been many reports that treat these mutations collectively as major uncommon mutations (1,2). It is known that patients with these gene mutations respond to second-generation EGFR- tyrosine kinase inhibitors (TKIs), but patients with Exon 20 insertions do not respond to EGFR-TKIs (3,4). At present, the existence of patients with many compound mutations with common or uncommon EGFR mutations has been recognized (4). Not only are they rare, but they are also genetically heterogeneous populations, and their responses to therapeutic drugs are not the same. As such, there are not many studies investigating patient backgrounds, such as age and smoking, in detail (5-19). In particular, only a few studies have shown information on more than 100 patients with uncommon mutations (6-8,11,13,15,18).

In view of this, we conducted this study to clarify clinical characteristics, with particular focus on age and smoking history, of NSCLC patients with uncom- mon EGFR mutations.

## MATERIALS and METHODS

The medical records of all NSCLC patients diagnosed at 14 medical institutions in our prefecture from July 2002 to December 2023 were examined. Based on the World Health Organization classification, the pathological diagnosis of each NSCLC patient was made (20). Before starting treatment, all patients underwent TNM classification using head computed tomography or magnetic resonance imaging, bone or positron emission scan, and abdominal ultrasound and/or computed tomography (21). At the time of NSCLC diagnosis, the following patient background characteristics were investigated: Sex, age, Eastern Cooperative Oncology Group performance status (PS), clinical stage, presence of EGFR mutation and EGFR mutation subtype. The ‘number of cigarettes smoked per day’ and ‘years of smoking’ were also investigated. The product of these indices was used as the smoking index (22,23).

For statistical analyses, the Chi-squared test was used to test for differences in proportions. The Mann- Whitney U test was used to compare values between two unmatched groups, such as patient age and smoking index. Correlations were examined using the Spearman correlation coefficient. A P-value less than 0.01 was considered to indicate a significant difference.

This study was approved by the Institutional Review Board of University of Tsukuba Mito Medical Center/ Mito Kyodo General Hospital (NO-23-53) and by each institute that participated in this study.

## RESuLTS

**Characteristics of Patients**

During the study period, clinical information on 158 patients with uncommon EGFR mutations was col- lected from 14 institutions. Median age of these patients was 72 years (range, 35-92 years), and there were 86 male and 72 female patients. There were 153 patients with adenocarcinoma and five patients with other histological types. The clinical stage was IA-IIIC in 83 patients, and IVA-B in 75 patients. With regard to PS, 137 patients had PS 0-1, and 21 patients had PS 2-4. There were 98 patients with major uncommon mutations, *G719X*, *L861Q*, and *S768I*, 41 with com- pound mutations, and 19 with Exon 20 insertions. Shows the age distribution of all patients Figure 1. The highest number of patients were in their 70s. There were 25 patients aged up to 59 years, and 47 patients aged 75 years or older. There was a significant differ- ence between the proportion of patients aged up to 59 years and that aged 75 years and older (p= 0.0046).

## Comparison Among uncommon EGFR Mutations

A comparison of patient background factors was per- formed with patients with three major mutations, *G719X*, *L861Q*, and *S768I*, as Group 1, patients with compound mutations as Group 2, and patients with Exon 20 insertions as Group 3. Patient background

factors among the three groups are shown in Table 1. There were no significant differences in age, sex his- tology, clinical stage, or PS among the three groups. In addition, we focused on smoking and compared the percentage of non-smokers, the percentage of light smokers (smoking index of 100 or less), and the smoking index, but there were no significant differ- ences among the three groups.

## Correlation and Comparison between Age and Smoking Index in the Three Groups Due to uncommon EGFR mutations

Figure 2-A shows the correlation between smoking index and age in all 158 patients. There was no sig- nificant correlation between smoking index and age in these patients (Spearman’s rank correlation co- efficient p= 0.9059, p= 0.009). Next, we investigated the correlation between smoking index and age among the 88 smokers. The results are shown in Figure 2-B. For smokers only, there was a significant correlation between smoking index and age (Spearman’s rank correlation co-efficient p= 0.0002, p= 0.397).

Patients were divided into non-smokers and smokers, and their age distributions are shown in Figures 3-A and B. In both groups, 70 non-smokers and 88 smokers, the most modal value for age was in the 70s. For non-smokers, the peak was at ages 70-74,

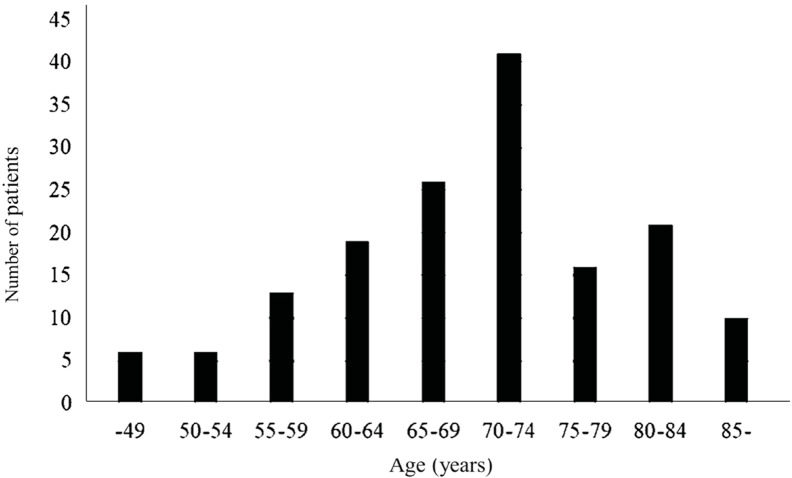

**Figure 1.** Age distribution of all 158 NSCLC patients with uncommon EGFR mutation (86 male patients and 72 female patients).

**Table:** 

**Table 1.** Comparison of patient background factors among the three groups of patients: Group 1 (major mutations, *G719X*, *L861Q*, and *S768I*), Group 2 (compound mutations), and Group 3 (Exon 20 insertions)				
	**Group 1**	**Group 2**	**Group 3**	**p**
Number of patients	98	41	19	
Age, median (range) years	72 (47-92)	68 (38-92)	73 (35-89)	0.2664
Sex				
Male	50 (51.0%)	23 (56.1%)	13 (68.4%)	0.3670
Female	48 (49.0%)	18 (43.9%)	6 (31.6%)	
Pathology				
AD	95 (96.9%)	39 (95.1%)	19 (100%)	0.6014
Others	3 (3.1%)	2 (4.9%)	0 (0%)	
Stage				
IA-IIIC	53 (54.1%)	20 (48.8%)	10 (52.6%)	0.8497
IVA-B	45 (45.9%)	21 (51.2%)	9 (47.4%)	
PS				
0-1	85 (86.7%)	36 (87.8%)	16 (84.2%)	0.9237
2-4	13 (13.3%)	5 (12.2%)	3 (15.8%)	
Non-smoker	45 (45.9%)	18 (43.9%)	7 (36.8%)	0.7653
Smoker	53 (54.1%)	23 (56.1%)	12 (63.2%)	
Non-light-smoker (SI< 100)	48 (49.0%)	19 (46.3%)	9 (47.4%)	0.8583
Smoker	50 (51.0%)	22 (53.7%)	10 (52.6%)	
SI, median (range)	130 (0-2400)	180 (0-1600)	150 (0-1500)	0.6227
AD: Adenocarcinoma, PS: Performance status, SI: Smoking index.

**Figure 2.** Correlation between smoking index and age in all 158 NSCLC patients **(A)**. There was no significant correlation between smoking index and age in these patients (Spearman’s rank correlation co-efficient p= 0.9059, p= 0.009). Correlation between smok- ing index and age in 88 NSCLC patients with smoking habit **(B)**. There was a significant correlation between smoking index and age (Spearman’s rank correlation co-efficient p= 0.0002, p= 0.397).

and for smokers, it was at ages 65-69. There was a significant difference in the age distribution of the non-smoker and smoker groups (p= 0.0192, Chi- squared test).

## DISCuSSION

This study confirmed the following results: The median age of the 158 patients with EGFR uncommon mutations was 72 years. Regarding the age distribution

of all patients, the most modal value for age was in the 70s. There was a significant difference between the proportion of the patients aged up to 59 years and the proportion of those aged 75 years or older. In 88 patients with a smoking habit, a significant correlation was found between the smoking index and age. Among non-smokers, there was a peak between ages 70 and 74, which was older than the peak among smokers.

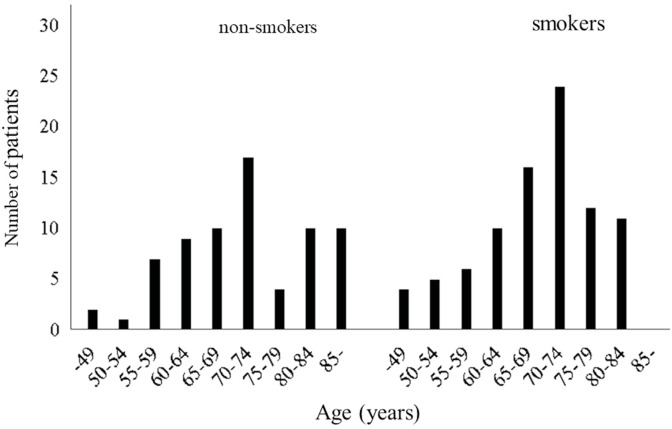
 **A B
**

**Figure 3.** Age distribution of 70 NSCLC patients without smoking habit **(A)** and 88 NSCLC with smoking habit **(B)**. Significant differ- ence in the age distribution between these two groups of patients (p= 0.0192, Chi-squared test).

Most studies to date on patients with uncommon EGFR mutations have focused on stage III-IV patients (5-7,10,12-17), and very few reports have included data on patients at all stages (9,19). Furthermore, the number of patients evaluated in these previous stud- ies has been very small; 26 and 40 patients, respec- tively (9,19). In past surveys of patients with stage III-IV disease and uncommon EGFR mutations, the proportion of female patients was 37%-75% (5-7,9- 19), and the proportion of patients with PS 0-1 was 60%-100% (6,9,10,12-17). The proportion of patients with adenocarcinoma was 16.7% in a study of 291 patients by Evans et al. (11), but other studies have generally reported proportions over 90% (5,7,10-17). In previous studies involving more than 100 patients, uncommon mutations have been classified into three groups: Major uncommon mutations, *G719X*, *L861Q*, and *S768I*; compound mutations; and Exon 20 inser- tions (6,7,11,13,15,18). Our study involved a rela- tively large number of patients, including patients at all stages of NSCLC from stage IA to stage IVB. In this survey, 42.4% were women, 86.7% were patients with PS 0-1, and 96.8% were adenocarcinoma patients. Focusing on uncommon mutation subtypes, 98 patients (62%) had major, 41 (25.9%) had com- pound, and 19 (12%) had Exon 20 insertions. It is

known that the positive rate of EGFR mutations in NSCLC differs between Asians and Caucasians (24), and it was necessary to confirm these background factors. However, these results were not significantly different from previous studies (5-19).

In previously conducted EGFR-TKI clinical trials, median age of the patients with uncommon EGFR mutations was 58-64 years (6,12,14). In a recent TKI clinical trial of over 40 patients with uncommon EGFR mutations, median age has been found as 72 years (19). On the other hand, in most studies in clinical practice except one (11), median age has been found as 59-68 years (5,7-11,13,15-18). The exception is a study of 291 patients in the United Kingdom by Evans et al., in which the average age is

70.1 years (11). The results from clinical practice from Evans et al. and our study suggest that even patients older than 70 years might harbor uncommon EGFR mutations (11). Not conducting a search for driver genes in NSCLC due to advanced age should be avoided, as this might limit treatment options.

In studies conducted so far, the proportion of smokers among patients with uncommon EGFR mutations has been found as 44%-59% in clinical trials (12,14) and as 48%-69% in clinical practice, except for one study

from India involving 40 patients, which has shown a non-smoking rate of 83% (7,9,10,13,15-18). In the present study, the proportion of non-smokers was 44.3%. Although it has been reported that the pro- portion of smokers is higher in patients with uncom- mon EGFR mutations than in patients with common mutations, to the best of our knowledge, there have been no reports that have considered both smoking history and age (13). The results of this study show that in both groups, 70 smokers and 88 non-smokers, the most modal value for age was in the 70s. On the other hand, there was a significant difference in age distribution between the non-smoker and smoker groups. In other words, the number of patients increased up to the age of 74 in both non-smoking and smoking groups, and after that, the distribution showed a difference between the two groups. A sig- nificant correlation was found between age and smoking index in patients with smoking history. It has been speculated that this is not simply due to an increase in the number of years of smoking, but that there may be some other cause that remains unknown.

Although the above novel findings were obtained, this study has some limitations. We used several testing methods for EGFR mutations, but comparisons could not be made because the testing methods were not integrated due to the multicenter nature of the study, and there was no information on EGFR gene- negative patients who were treated around the same time. In addition, the study period was long because it was intended to collect a large number of patients. However, there are few reports that have investigated more than 150 patients, and we do believe that the information obtained might be useful for the future medical treatment of patients with uncommon EGFR mutations.

## CONCLuSION

The implementation of driver gene testing for NSCLC is expected to provide important information for selecting treatment options tailored to the patient. Therefore, even in NSCLC patients who are elderly or who have a history of smoking, although it is unclear whether EGFR mutations are common or uncommon, EGFR gene testing should be performed in case an EGFR mutation is present.

## Acknowledgment

We would like to thank the researchers at the following facilities for their cooperation in this study:

Hitachi General Hospital, Hitachinaka General Hospital, Ibarakihigashi Hospital, Tsukuba Medica Center Hospital, Tsuchiura Kyodo General Hospital, Ibaraki Medical Center-Tokyo Medical University.

**Ethical Committee Approval:** This study was obtained from General Hospital Mito Kyodo Hospital Director of Hospital Ethics Committee (Decision no: 23-53, Date: 13.12.2023).

## CONFLICT of INTEREST

The authors declare that they have no conflict of interest.

## AuTHORSHIP CONTRIBuTIONS

Concept/Design: YM, SO, KM, HS Analysis/Interpretation: YM, SO, KM, HS

Data acqusition: YM, MT, TK, TL, TN, TS, KM, RN, KI, TS

Writing: YM, KM, HS Clinical Revision: HS Final Approval: HS, NH

## REFERENCES

1. *
  Attili I, Passaro A, Pisapia P, Malapelle U, de Marinis F. Uncommon EGFR compound mutations in non-small cell lung cancer (NSCLC): A systematic review of available evidence. Curr Oncol 2022; 29: 255-66. https://doi.org/10.3390/curroncol29010024
  *
2. *
  John T, Taylor A, Wang H, Eichinger C, Freeman C, Ahn MJ. Uncommon EGFR mutations in non-small-cell lung can- cer: A systematic literature review of prevalence and clinical outcomes. Cancer Epidemiol 2022; 76: 102080. https://doi.org/10.1016/j.canep.2021.102080
  *
3. *
  Dorta-Suárez M, de Miguel M, Amor-Carro O, Calderón JM, González-Ortega M, Rodríguez-Abreu D. The state of the art of EGFR exon 20 insertions in non-small cell lung cancer: Diagnosis and future perspectives. Cancer Treat Rev 2023; 124: 102671. https://doi.org/10.1016/j.ctrv.2023.102671
  *
4. *
  Pretelli G, Spagnolo CC, Ciappina G, Santarpia M, Pasello G. Overview on therapeutic options in uncommon EGFR mutant non-small cell lung cancer (NSCLC): New lights for an unmet medical need. Int J Mol Sci 2023; 24: 8878. https://doi.org/10.3390/ijms24108878
  *
5. *
  Heigener DF, Schumann C, Sebastian M, Sadjadian P, Stehle I, Märten A, et al. Afatinib Compassionate Use Consortium (ACUC). Afatinib in non-small cell lung can- cer harboring uncommon EGFR Mutations pretreated with reversible EGFR inhibitors. Oncologist 2015; 20: 1167-74. https://doi.org/10.1634/theoncolo-gist.2015-0073
  *
6. *
  Yang JC, Sequist LV, Geater SL, Tsai CM, Mok TS, Schuler M, et al. Clinical activity of afatinib in patients with advanced non-small-cell lung cancer harbouring uncom- mon EGFR mutations: A combined post-hoc analysis of LUX-Lung 2, LUX-Lung 3, and LUX-Lung 6. Lancet Oncol 2015; 16: 830-8. https://doi.org/10.1016/S1470-2045(15)00026-1
  *
7. *
  Tu HY, Ke EE, Yang JJ, Sun YL, Yan HH, Zheng MY, et al. A comprehensive review of uncommon EGFR mutations in patients with non-small cell lung cancer. Lung Cancer 2017; 114: 96-102. https://doi.org/10.1016/j.lung-can.2017.11.005
  *
8. *
  Wu SG, Chang YL, Yu CJ, Yang PC, Shih JY. Lung adenocar- cinoma patients of young age have lower EGFR mutation rate and poorer efficacy of EGFR tyrosine kinase inhibi- tors. ERJ Open Res 2017; 3: 00092-2016. https://doi.org/10.1183/23120541.00092-2016
  *
9. *
  Frega S, Lorenzi M, Fassan M, Indraccolo S, Calabrese F, Favaretto A, et al. Clinical features and treatment outcome of non-small cell lung cancer (NSCLC) patients with uncommon or complex epidermal growth factor receptor (EGFR) mutations. Oncotarget 2017; 8: 32626-38. https://doi.org/10.18632/oncotarget.15945
  *
10. *
  Chantharasamee J, Poungvarin N, Danchaivijitr P, Techawatanawanna S. Clinical outcome of treatment of metastatic non-small cell lung cancer in patients harboring uncommon EGFR mutation. BMC Cancer 2019; 19: 701. https://doi.org/10.1186/s12885-019-5913-9
  *
11. *
  Evans M, O’Sullivan B, Smith M, Hughes F, Mullis T, Trim N, et al. Large-scale EGFR mutation testing in clinical prac- tice: Analysis of a series of 18,920 non-small cell lung cancer cases. Pathol Oncol Res 2019; 25: 1401-9. https://doi.org/10.1007/s12253-018-0460-2
  *
12. *
  Cho JH, Lim SH, An HJ, Kim KH, Park KU, Kang EJ, et al. Osimertinib for patients with non-small-cell lung cancer harboring uncommon EGFR mutations: A multicenter, open-label, phase II trial (KCSG-LU15-09). J Clin Oncol 2020; 38: 488-95. https://doi.org/10.1200/JCO.19.00931
  *
13. *
  Ko HW, Shie SS, Wang CW, Chiu CT, Wang CL, Yang TY, et al. Association of smoking status with non-small cell lung cancer patients harboring uncommon epidermal growth factor receptor mutation. Front Immunol 2022; 13: 1011092. https://doi.org/10.3389/fimmu.2022.1011092
  *
14. *
  Li HS, Yang GJ, Cai Y, Li JL, Xu HY, Zhang T, et al. Dacomitinib for advanced non-small cell lung cancer patients harboring major uncommon EGFR alterations: A dual-center, single-arm, ambispective cohort study in China. Front Pharmacol 2022; 13: 919652. https://doi.org/10.3389/fphar.2022.919652
  *
15. *
  Popat S, Hsia TC, Hung JY, Jung HA, Shih JY, Park CK, et al. Tyrosine kinase inhibitor activity in patients with NSCLC harboring uncommon EGFR mutations: A retrospective international cohort study (UpSwinG). Oncologist 2022; 27: 255-65. https://doi.org/10.1093/oncolo/oyac022
  *
16. *
  Ullas B, Shrinidhi N, Mansi S, Narayan S, Parveen J, Surender D, et al. All EGFR mutations are (not) created equal: Focus on uncommon EGFR mutations. J Cancer Res Clin Oncol 2023; 149: 1541-9. https://doi.org/10.1007/s00432-022-04033-x
  *
17. *
  Bar J, Peled N, Schokrpur S, Wolner M, Rotem O, Girard N, et al. Uncommon EGFR mutations: International case series on efficacy of osimertinib in real-life practice in first- line setting (UNICORN). J Thorac Oncol 2023; 18: 169-80. https://doi.org/10.1016/j.jtho.2022.10.004
  *
18. *
  Wang C, Zhao K, Hu S, Dong W, Gong Y, Xie C. Clinical outcomes of afatinib versus osimertinib in patients with non-small cell lung cancer with uncommon EGFR muta- tions: A pooled analysis. Oncologist 2023; 28: e397-e405. https://doi.org/10.1093/oncolo/oyad111
  *
19. *
  Okuma Y, Kubota K, Shimokawa M, Hashimoto K, Kawashima Y, Sakamoto T, et al. Tokyo Cooperative Oncology Group (TCOG). First-line osimertinib for previ- ously untreated patients with NSCLC and uncommon EGFR mutations: The UNICORN phase 2 nonrandomized clinical trial. JAMA Oncol 2024; 10: 43-51. https://doi.org/10.1001/jamaoncol.2023.5013
  *
20. *
  WHO Classification of Tumours Editorial Board. World Health Organization Classification of Tumours, Thoracic Tumours 5th ed. IARC Press, 2021.
  *
21. *
  Goldstraw P, Chansky K, Crowley J, Rami-Porta R, Asamura H, Eberhardt WE, et al. International Association for the Study of Lung Cancer Staging and Prognostic Factors Committee, Advisory Boards, and Participating Institutions, International Association for the Study of Lung Cancer Staging and Prognostic Factors Committee Advisory Boards and Participating Institutions The IASLC lung can- cer staging project: Proposals for revision of the TNM stage groupings in the forthcoming (eighth) edition of the TNM classification for lung cancer. J Thorac Oncol 2016; 11: 39-51. https://doi.org/10.1016/j.jtho.2015.09.009
  *
22. *
  Maeshima AM, Tochigi N, Tsuta K, Asamura H, Matsuno Y. Histological evaluation of the effect of smoking on periph- eral small adenocarcinomas of the lung. J Thorac Oncol 2008; 3: 698-703. https://doi.org/10.1097/JTO.0b013e31817c60ae
  *
23. *
  Nakamura R, Inage Y, Tobita R, Yoneyama S, Numata T, Ota K, et al. Sarcopenia in resected NSCLC: Effect on postoperative outcomes. J Thorac Oncol 2018; 13: 895-903. https://doi.org/10.1016/j.jtho.2018.04.035
  *
24. *
  Batra U, Biswas B, Prabhash K, Krishna MV. Differential clinicopathological features, treatments and outcomes in patients with Exon 19 deletion and Exon 21 L858R EGFR mutation-positive adenocarcinoma non-small-cell lung cancer. BMJ Open Respir Res 2023; 10: e001492. https://doi.org/10.1136/bmjresp-2022-001492
  *

